# A bibliometric analysis of NLRP3 inflammasome in acute lung injury/acute respiratory distress syndrome from 2010 to 2021

**DOI:** 10.3389/fimmu.2022.1053658

**Published:** 2022-12-21

**Authors:** Zhuoran Xiao, Song Hu, Wenting Xu, Sheng Wang, Wei Mo, Huimin Deng, Juan Wei, Hao Yang, Wenyu Zhou, Quanfu Li, Huanping Zhou, Xin Lv

**Affiliations:** Department of Anesthesiology, Shanghai Pulmonary Hospital, School of Medicine, Tongji University, Shanghai, China

**Keywords:** NLRP3 inflammasome, acute lung injury, acute respiratory distress syndrome, bibliometrics, CiteSpace

## Abstract

**Background:**

Nod-like receptor family pyrin domain containing 3 (NLRP3) inflammasome is essential in the pathogenesis of acute respiratory distress syndrome (ARDS), a fatal clinical syndrome that deteriorated from acute lung injury (ALI). This bibliometric study aims to offer a thorough insight into the scientific output about NLRP3 inflammasome in ALI/ARDS and explore the intellectual base, developing trajectory and emerging trends.

**Methods:**

We retrieved the literature from 2010 to 2021 from Science Citation Index Expanded (SCIE) database. Bibliometrix (3.1.4) R package and CiteSpace (5.8.R3) were used for further analysis and visualization.

**Results:**

A total of 508 English articles and reviews published from 2010 to 2021 were identified. The annual number of publications presented a rapidly developing trend especially in recent years. Among all the 42 countries, China was the most productive and most cited country, while the USA had the greatest impact. Peter A. Ward from the USA was the most productive corresponding author, and 4 of these top 10 corresponding authors were from China. The most cited reference was written by Ahmed (2017) of Zhejiang University in China. The Journal of Immunology had highest citation count and G-index. Furthermore, the major disciplines of research front have drifted from “Medicine, Medical, Clinical” to “Molecular, Biology, Immunology” over the past 12 years. In the co-occurring network, the terms “acute lung injury,” “NLRP3 inflammasome,” “interleukin-1β,” “NF-κB,” and “NLRP3 activation” occurred most frequently, while in burst detection, “oxidative stress” had the highest burst strength. Co-citation network revealed that Cluster 2 “virus infection” was the most active area, including the most citation bursts. Cluster 0 “severe COVID-19” and Cluster 1 “dual inhibitor PTUPB” were emerging themes in recent years, and they involved the largest number of publications.

**Conclusions:**

This bibliometric analysis revealed a rapid growth trend of the relatively novel topic: NLRP3 inflammasome in ALI/ARDS. China was the largest contributor, while the USA offered the most landmark papers. The major disciplines of research front drifted from “Medicine, Medical, Clinical” to “Molecular, Biology, Immunology.” In recent years, studies about the role of NLRP3 in COVID-19-associated ALI/ARDS and oxidative stress became hot spots.

## 1 Introduction

Acute respiratory distress syndrome (ARDS), presented as the severe phase of acute lung injury (ALI), is a clinical syndrome featured with acute hypoxemic respiratory failure accompanied by bilateral infiltrates on chest image, which is not entirely interpreted by cardiac failure or fluid overload (1). It is often precipitated by certain predisposing factors such as pneumonia, aspiration of gastric contents, pancreatitis, shock, non-pulmonary sepsis, or severe trauma (2). Pathologically, it is characterized by diffuse alveolar damage, alveolar epithelial and pulmonary microvascular endothelial cell injury, and accumulation of inflammatory edematous fluid (3–5). Nod-like receptor family pyrin domain containing 3 (NLRP3) inflammasome, a kind of intracellular protein complex activated by a wide spectrum of stimuli such as microbial infection, damaged lysosomes and Ca^2+^ flux, is reported to be activated in ALI of different types and is thought to be essential in pathogenesis (6–10). NLRP3 inflammasome is closely associated with the activation of inflammatory caspases, which afterwards trigger the release of proinflammatory cytokines like interleukin-1β (IL-1β), IL-18, IL-6, and tumor necrosis factor (TNF), and induce pyroptosis (9–11). In addition, NLRP3 inflammasome and related proinflammatory cytokines also play an important role in coronavirus disease 2019 (COVID-19)-associated ALI/ARDS, showing potential of targeting NLRP3 inflammasome in treatment (11–14).

Bibliometrics is an interdisciplinary method to quantitatively analyze the feature and structure of a set of information. This method has been popularly used in different areas because of its advancement, availability, and accessibility (15). Bibliometric analysis can help describe the data group in a comprehensive overview, making it easier to understand the intellectual structure, thus exploring the development path and emerging trends of the domain. This study aims to depict the characteristics and offer a thorough insight into the documents data about NLRP3 inflammasome in ALI/ARDS and further dig into the intellectual base, developing trajectory and emerging trends.

## 2 Materials and methods

### 2.1 Data preparation

Original bibliographic data were downloaded from Science Citation Index Expanded (SCIE) of Web of Science Core Collection (WoSCC) developed by Thomson Scientific. To reduce deviation and enhance credibility, two observers conducted the literature retrieval independently on the same date (29 March 2022), and the agreement exceeded 0.9, indicating relative reliability (16).

Under the reference of MeSH, the final query terms were as follows:

TS=[(NLRP3)OR(NLPR3 inflammasome) OR (NLR Family Pyrin Domain Containing 3 Protein) OR (Nod-like receptor protein 3 inflammasome)] AND TS=[(ALI) OR (ARDS) OR (acute lung injury) OR (respiratory distress syndrome) OR (acute respiratory distress syndrome)]

**Figure 1:**
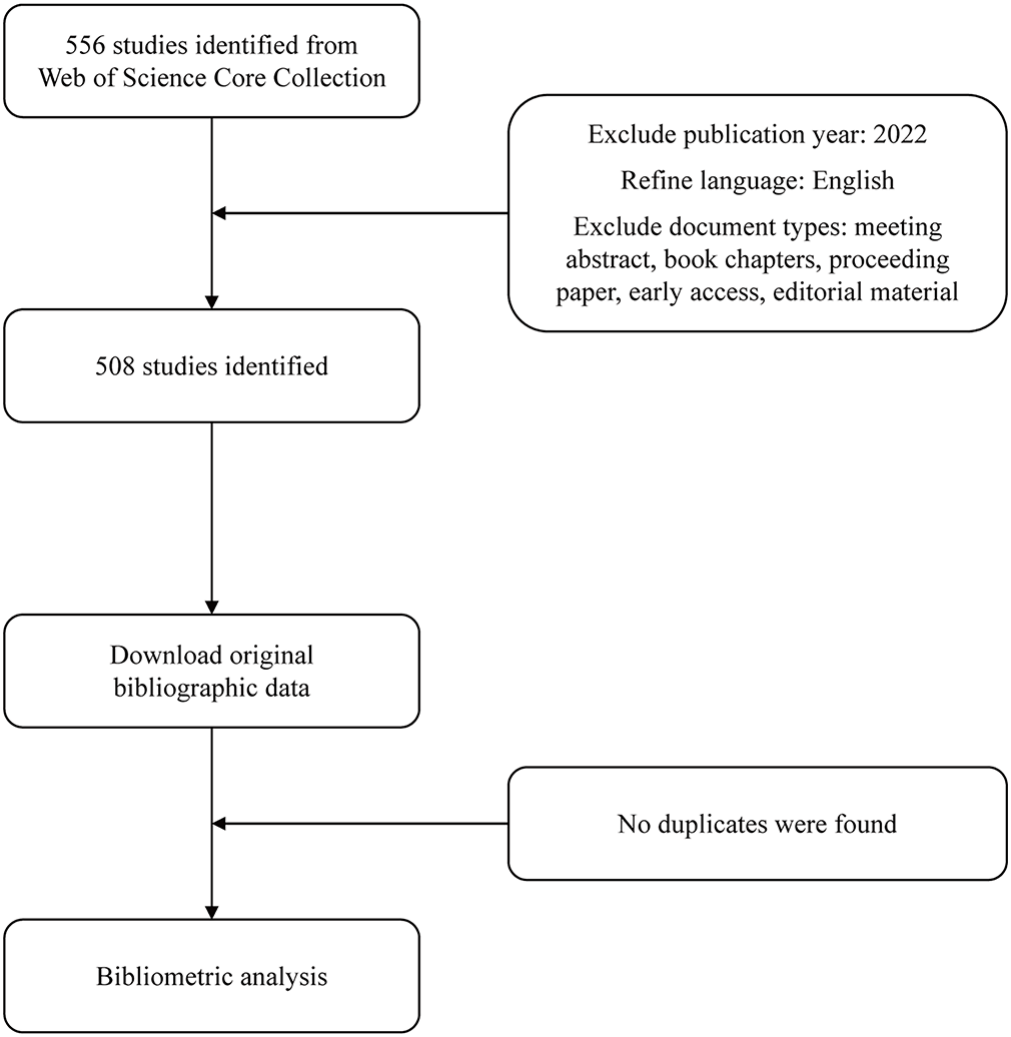
Flowchart of the data preparation process.

After the basic searching, we found that the earliest publications about NLRP3 in ALI/ARDS collected in WoSCC were published in 2010, so the publication year was set from 2010 to 2021. Only “Article” and “Review Article” written in English were included in the subsequent bibliometric analysis. Ultimately, we included 508 records altogether, and no duplicates were found (**Figure 1**).

### 2.2 Analysis method

As massive and complex the data can be, we conducted this bibliometric analysis roughly following the path from the general to details. Analyzed information included countries, affiliations, authors, publication year, journal, references, and keywords. Data collected were later imported into Bibliometrix (3.1.4) and CiteSpace (5.8.R3) for further analysis.

Bibliometrix is an open-source tool programmed in R language and designed by Massimo Aria and Corrado Cuccurullo (17). This R package can be used to perform comprehensive analysis.

CiteSpace is a Java application that allows visualization of the evolution of a knowledge domain’s collaborative networks. The software simplifies the search for intellectual turning points to a search for visually salient features in the visualized work. With data loaded, CiteSpace uses a time slicing technique to model the intellectual structure of its domain through synthesizing an overview network based on time series. Synthesized network is further divided into different clusters. These visualized networks are based on the theory of co-citation, which posits that two papers share a co-citation relationship when they are cited together by another document (18, 19). In CiteSpace, betweenness centrality is a useful metric to identify connective references in the network. It measures the extent to which the nodes participate in connections between different groups of nodes. It is studied that nodes with high betweenness centrality scores tend to point to valuable boundary spanning potentials (20). Burst detection can help find references with an abrupt rise of citations, thus identifying the most active areas of research. If a cluster has several nodes with strong citation bursts, then we can say it captures an emerging trend. The sigma score combines both the above two metrics to make a comprehensive evaluation (21).

Alternative metrics enable people to evaluate the global performance of a set of articles from different aspects. The most basic measure of an article, journal, or region’s influence is the number of citations to it. Citation count is also related to the number of articles and publication year and not feasible to be used independently (22, 23). “H” was first proposed by Jorge Hirsch as a useful index to evaluate the contribution and impact of a scientist, indicating that the researcher has written H papers and each paper has been cited at least H times (24). H-index can be applied not only for the influence of a single researcher but also for any publication set with enough quantity (25). However, H-index can be limited and inaccurate when it comes to small article set. G-index is presented as an improvement of H-index. G-index is the largest rank (where papers are arranged in decreasing order of the citation count) such that the first g papers have at least g^2^ citations altogether (26). It considers more of the performance of well-cited articles while keeping all the good properties of H-index, better representing overall achievements than H-index (26). At journal level, Journal Impact Factor (JIF) reported by Thomson Reuters Journal Citation Reports (JCR) has been considered as one of the leading proxies and widely applied for evaluating journals regardless of their size (23, 27). Correlation is shown between high scientific level journals and that with high JIF (28).

## 3 Results

### 3.1 Global overview

Based on the search strategy, we included 508 papers (419 articles and 89 reviews) with a time span of 12 years (2010–2021). The total number of citations was 12,534; moreover, 21 publications received more than 100 times of citation. The general H-index was 52. From the overall perspective, 2,706 authors from 42 regions and countries published literature in 208 journals globally.

### 3.2 Annual trend

**Figure 2:**
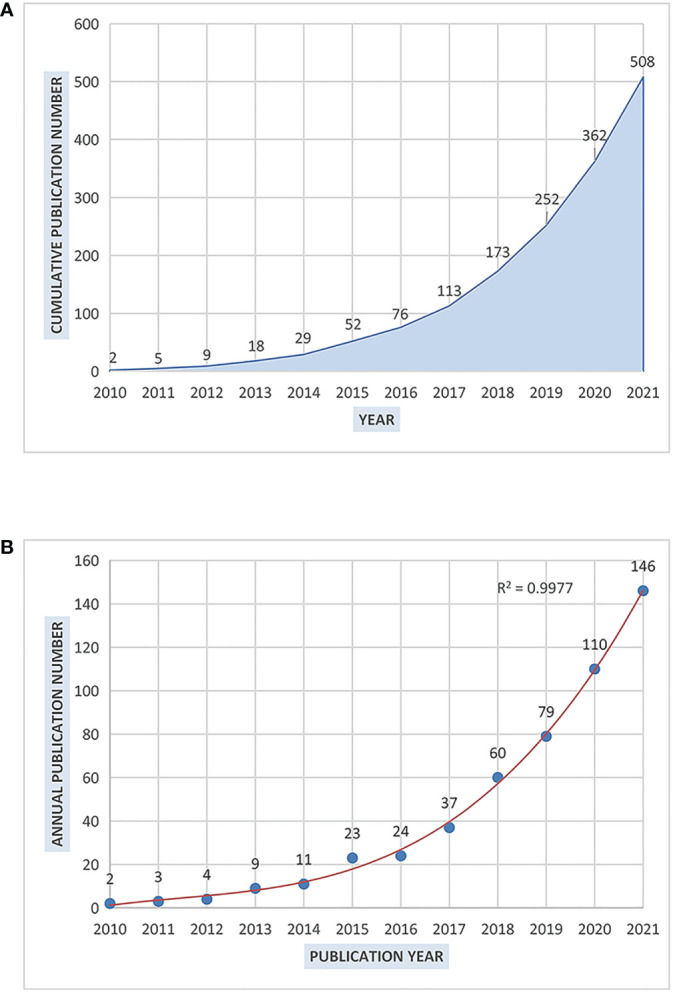
The increasing tendency of the number of literatures about NLRP3 in ALI/ARDS. **(A)** The cumulative number of publications from 2010 to 2021. **(B)** The annual number of publications from 2010 to 2021 and the polynomial fitting curve (R^2^ = 0.9977).

As is shown in **Figure 2A**, papers about NLRP3 in ALI/ARDS displayed an increasing tendency ever since showing up. **Figure 2B** presents the annual number of publications and the polynomial fitting curve (cubic) with the correlation coefficient R^2^ as 0.9977, indicating high degree of correlation between the annual number of publications and the publication year. The annual growth rate was calculated as 47.7% by Bibliometrix. We can see that the growth speed has also become faster over time.

### 3.3 Overall contribution

#### 3.3.1 Country level

A total of 42 countries were involved in the knowledge production, but over 85% of the publications were contributed by researchers from the top 2 active countries (China and the USA). China was the most productive and most cited country in this field (322 papers/63.39%, 6,018 citations), followed by the USA (114 papers/22.44%, 4,527 citations). We depicted the top 10 prolific countries in **Table 1**. The USA possessed the greatest impact in this field with the highest H-index (41) and G-index (63), closely followed by China (H-index 39 and G-index 61) in second. As for the other top countries, it is difficult to evaluate their contributions expressly with H-index as the small volume of articles, but G-index still worked well to represent the overall performances. Germany, Italy, South Korea, and Canada had relatively high achievement with G-index of 33, 18, 15, and 15, respectively.

**Table 1:** The top 10 prolific countries.

Rank	Country	Documents (share)	Citations	H-index	G-index
1	China	322 (63.39%)	6,018	39	61
2	USA	114 (22.44%)	4,527	41	63
3	Germany	17 (3.35%)	1,122	12	33
4	Italy	13 (2.56%)	345	10	18
5	Iran	13 (2.56%)	124	6	11
6	England	12 (2.36%)	202	8	14
7	India	11 (2.17%)	61	4	7
8	South Korea	9 (1.77%)	233	7	15
9	Australia	8 (1.57%)	174	6	13
10	Canada	8 (1.57%)	225	7	15

#### 3.3.2 Institutional level

**Table 2** lists the details of the top 10 most active institutions in the knowledge production of NLRP3 in ALI/ARDS. China was the main country that these institutions affiliated to (7 institutions), except for League of European Research Universities (LERU), Pennsylvania Commonwealth System of Higher Education (PCSHE), and University of California (UNIV CALIF). Among the top 10 affiliations, although LERU was not very productive, it had the largest number of citations (656) and the highest G-index (25), showing potential impact in this area and high quality of publications. In addition, Central South University (CENT S UNIV), Jilin University (JILIN UNIV), PCSHE, and Fudan University (FUDAN UNIV) also showed good performances in the citation count, H-index, and G-index.

**Table 2:** The top 10 active affiliations.

Rank	Institution	Country/Region	Documents	Citations	H-index	G-index
1	Central South University	China	24	578	15	24
2	Shanghai Jiao Tong University	China	19	364	11	19
3	Fudan University	China	18	400	9	20
4	Jilin University	China	18	623	13	24
5	Southern Medical University	China	15	295	8	17
6	League of European Research Universities	Europe	12	656	11	25
7	Nanjing Medical University	China	12	219	9	14
8	Pennsylvania Commonwealth System of Higher Education	USA	11	409	8	20
9	Shandong University	China	11	128	6	11
10	University of California	USA	11	203	8	14

#### 3.3.3 Author level

**Table 3** presents the top 10 most productive corresponding authors in delivering articles about NLRP3 in ALI/ARDS. Four of these top 10 corresponding authors were from China. Peter A. Ward, the most productive corresponding author from the group of University of Michigan in the USA, wrote pioneering works and got remarkably high citations (7 documents, 399 citations, G-index 19). It is interesting to note that Cha-xiang Guan and Yong Zhou were from the same institution: Central South University in China. In addition, although some researchers only published one article in this field, they acquired high citations, for example, Ahmed, Luo, Namani, Wang, and Tang (1 article with 687 citations), and Kany, Vollrath, and Relja (1 article with 348 citations).

**Table 3:** The top 10 productive corresponding authors.

Rank	Corresponding Author	Affiliation	Country	Documents	Citations	G-index
1	Ward, Peter A.	University of Michigan	USA	7	399	19
2	Ci, Xinxin	Jilin University	China	6	363	19
3	Fan, Jie	University of Pittsburgh	USA	6	304	17
4	Guan, Cha-xiang	Central South University	China	6	194	13
5	Zhou, Yong	Central South University	China	4	100	10
6	Zhao, Min	China Medical University	China	4	98	9
7	Opitz, Bastian	Charite	Germany	3	235	15
8	Kuipers, Maria T.	University of Amsterdam	Netherlands	3	185	13
9	Takahashi, Masafumi	Jichi Medical University	Japan	3	58	7
10	Mahmoudian-Sani, Mohammad-Reza	Ahvaz Jundishapur University of Medical Sciences	Iran	3	30	5

#### 3.3.4 Journal level

**Figure 3:**
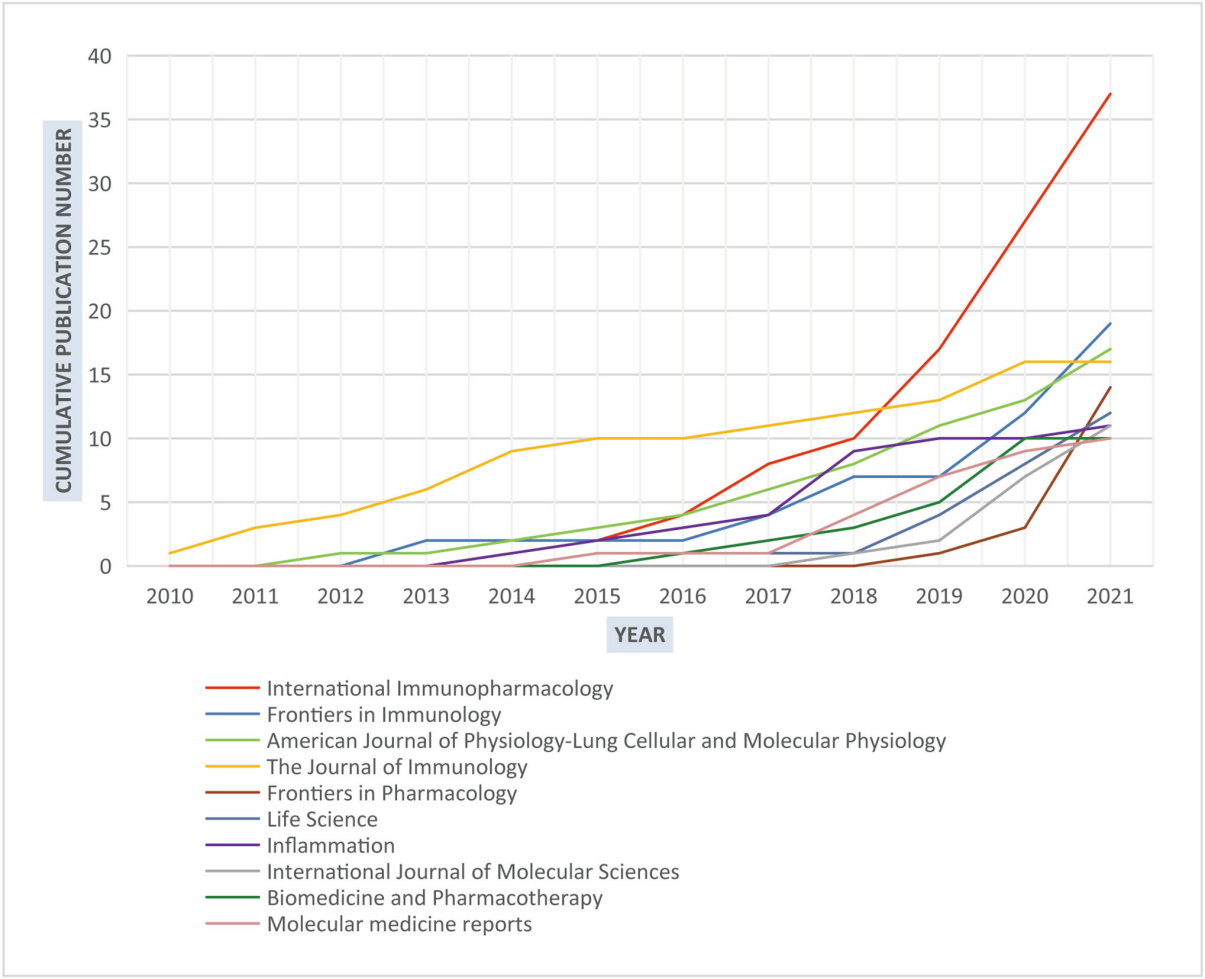
The growth trend of publications in the top 10 productive journals.

Generally, 208 journals participated in the publication of articles about NLRP3 in ALI/ARDS. As we display in **Figure 3**, *The Journal of Immunology* ranked first in total output over the first 9 years since 2010. However, when entering the 2019, the number of publications about NLRP3 in ALI/ARDS in *International Immunopharmacology* started to increase rapidly, which became the most productive source in this discipline (**Table 4**, 37 papers till 2021). With only 16 documents, *The Journal of Immunology* had much higher citation count (1,139) and G-index (33) than the others, indicating great importance to the knowledge contribution and classic articles possibly. The majority of these 10 productive journals had JIF >5. Meanwhile, we noted that several leading journals with high JIF were also involved in 208 journals: *Nature Reviews Immunology* (1 paper, JIF 108.555), *Immunity* (1 paper, JIF 43.474), *American Journal of Respiratory and Critical Care Medicine* (3 papers, 30.528), and *Nature Communications* (2 papers, JIF 17.694).

**Table 4:** The top 10 journals with the most publications.

Rank	Sources	Documents	Citations	JIF (2021)	H-index	G-index
1	International Immunopharmacology	37	544	5.714	14	22
2	Frontiers in Immunology	19	549	8.786	11	23
3	American Journal of Physiology-Lung Cellular and Molecular Physiology	17	338	6.011	9	18
4	The Journal of Immunology	16	1,139	5.426	12	33
5	Frontiers in Pharmacology	14	56	5.988	3	7
6	Life Science	12	191	6.78	8	13
7	Inflammation	11	295	4.657	9	17
8	International Journal of Molecular Sciences	11	530	6.208	7	23
9	Biomedicine and Pharmacotherapy	10	230	7.419	9	15
10	Molecular Medicine Reports	10	219	3.423	7	14

#### 3.3.5 Highly cited papers

We selected the top 15 highly cited papers and ranked them by the total number of citations in WOS Core Collection (**Table 5**), which included eight articles and seven reviews. Generally, they were published by dispersed research groups, but in country level, researchers from the USA contributed the most in these highly cited publications (seven papers).

**Table 5:** The top 15 papers with the highest number of citations.

Rank	Title	Author	Country	Journal (JIF 2021)	Date	Citations	Nc/year
1	Nrf2 signaling pathway: Pivotal roles in inflammation	Ahmed, SMU, et al	China	BBA-MOL BASIS DIS (JIF: 6.633)	Feb 2017	687	137.4
2	Cytokines in Inflammatory Disease	Kany, S, et al	Germany	INT J MOL SCI (JIF: 6.208)	Dec 2019	348	116
3	Extracellular ATP Is a Danger Signal Activating P2X(7) Receptor in Lung Inflammation and Fibrosis	Riteau, N, et al	France	AM J RESP CRIT CARE (JIF: 30.528)	Sep 2010	276	23
4	Why does COVID-19 disproportionately affect older people?	Mueller, AL, et al	USA	AGING-US (JIF: 5.955)	May 2020	273	136.5
5	Critical Role for the NLRP3 Inflammasome during Acute Lung Injury	Grailer, JJ, et al	USA	J IMMUNOL (JIF: 5.43)	Jun 2014	197	24.63
6	Xanthohumol ameliorates lipopolysaccharide (LPS)-induced acute lung injury *via* induction of AMPK/GSK3 beta-Nrf2 signal axis	Lv, HM, et al	China	REDOX BIOL (JIF: 10.787)	Aug 2017	184	36.8
7	The NLRP3 Inflammasome Is Differentially Activated by Pneumolysin Variants and Contributes to Host Defense in Pneumococcal Pneumonia	Witzenrath, M, et al	Germany	J IMMUNOL (JIF: 5.43)	Jul 2011	176	16
8	Extracellular histones in tissue injury and inflammation	Allam, R, et al	Germany	J MOL MED (JIF: 5.606)	May 2014	175	21.88
9	Melatonin and inflammation-Story of a double-edged blade	Hardeland, R	Germany	J PINEAL RES (JIF: 12.081)	Nov 2018	160	40
10	NLRP1-Dependent Pyroptosis Leads to Acute Lung Injury and Morbidity in Mice	Kovarova, M, et al	USA	J IMMUNOL (JIF: 5.43)	Aug 2012	153	15.3
11	Targeting the NLRP3 Inflammasome in Severe COVID-19	Freeman, TL, et al	USA	FRONT IMMUNOL (JIF: 8.787)	Jun 2020	141	70.5
12	Melatonin alleviates acute lung injury through inhibiting the NLRP3 inflammasome	Zhang, Y, et al	China	J PINEAL RES (JIF: 12.081)	May 2016	140	23.33
13	Inflammation and Fibrosis during Chlamydia pneumoniae Infection Is Regulated by IL-1 and the NLRP3/ASC Inflammasome	He, XB, et al	USA	J IMMUNOL (JIF: 5.43)	May 2010	126	10.5
14	Vimentin regulates activation of the NLRP3 inflammasome	dos Santos, G, et al	USA	NAT COMMUN (JIF: 17.694)	Mar 2015	124	17.71
15	The inflammasome in lung diseases	dos Santos, G, et al	USA	AM J PHYSIOL LUNG CELL MOL PHYSIOL (JIF: 6.011)	Oct 2012	120	12

**Figure 4:**
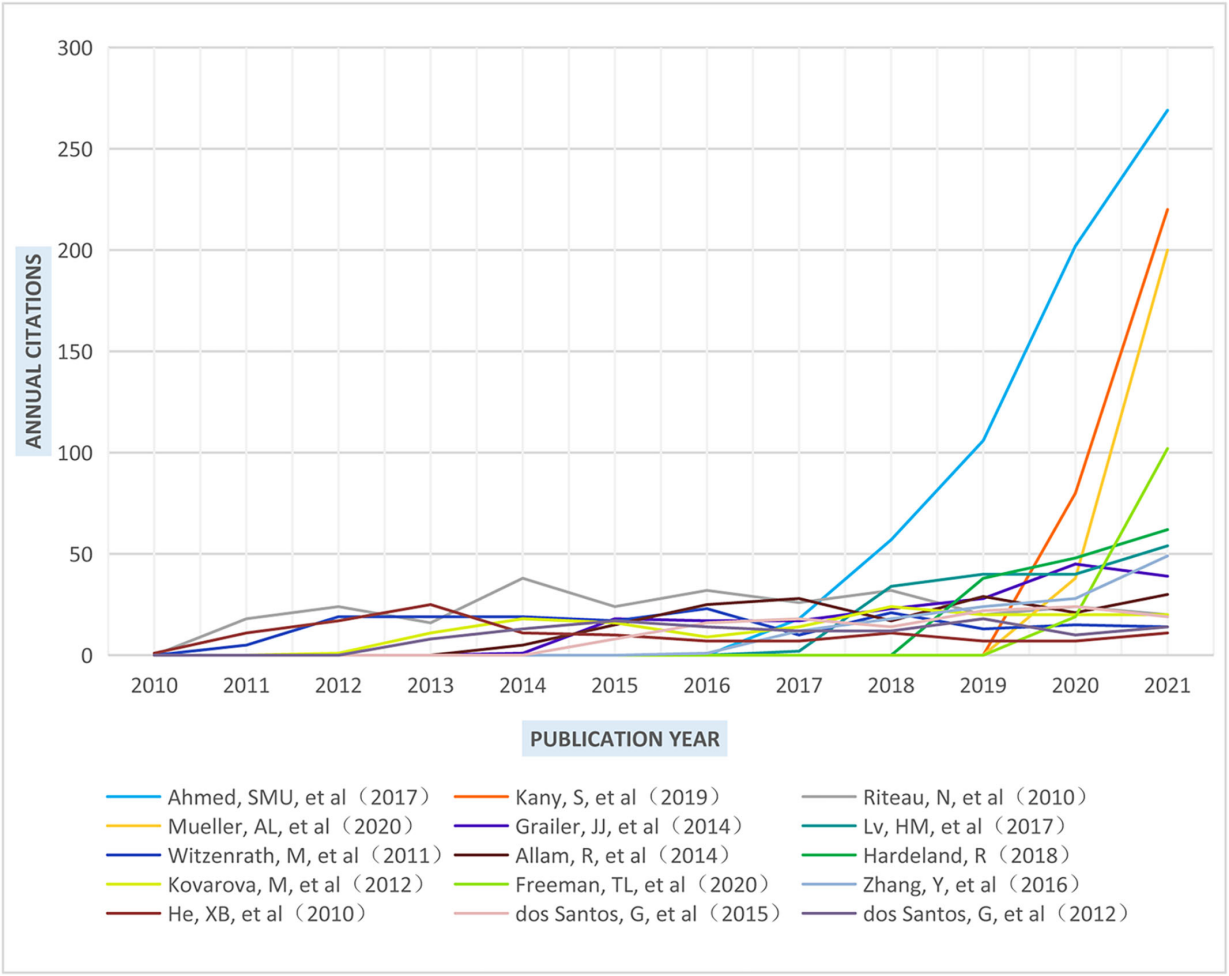
The annual number of citations of the top 15 highly cited papers.

The highest cited reference (687 citations, WOS Core), written by Ahmed (2017) from Zhejiang University in China, comprehensively discussed the landscape of Nrf2 signaling pathway including the protective role of some Nrf2-dependent drugs against ALI and its effects on the regulation of NLRP3 inflammasome (29). Furthermore, some publications delivered in recent years (rank 1, 2, 4, and 11) have been frequently used. These four landmark articles showed absolute advantages in 2021 citation count (**Figure 4**) compared with most of the earlier articles, polishing this discipline and showing future significance. In addition, the average number of citations per year (Nc/year) can reflect the importance and attention from other scholars without the disturbance of publication year. The above four articles also had the highest Nc/year, showing high quality and popularity since published. The article by Riteau (276 citations, Nc/year: 23, JIF: 30.528) from France received stable citations every year since published in 2010, indicating that it may act as the knowledge bases of this discipline to some extent.

It is also worth noting that two reviews with high average citation count in 2020 (rank 4 by Mueller and rank 11 by Freeman) both focused on COVID-19. Mueller’ s team discussed the molecular differences between the young and the old, which included the hyperactivation of NLRP3 by severe acute respiratory syndrome coronavirus 2 (SARS*-*CoV*-*2*)* antigens in older individuals (30). Freeman and Swartz reviewed the literature on pathogenesis of NLRP3 activation and severe COVID-19, concluding the necessity of investigation into NLRP3 inflammasome as a therapeutic target (12).

### 3.4 Major disciplines

**Figure 5:**
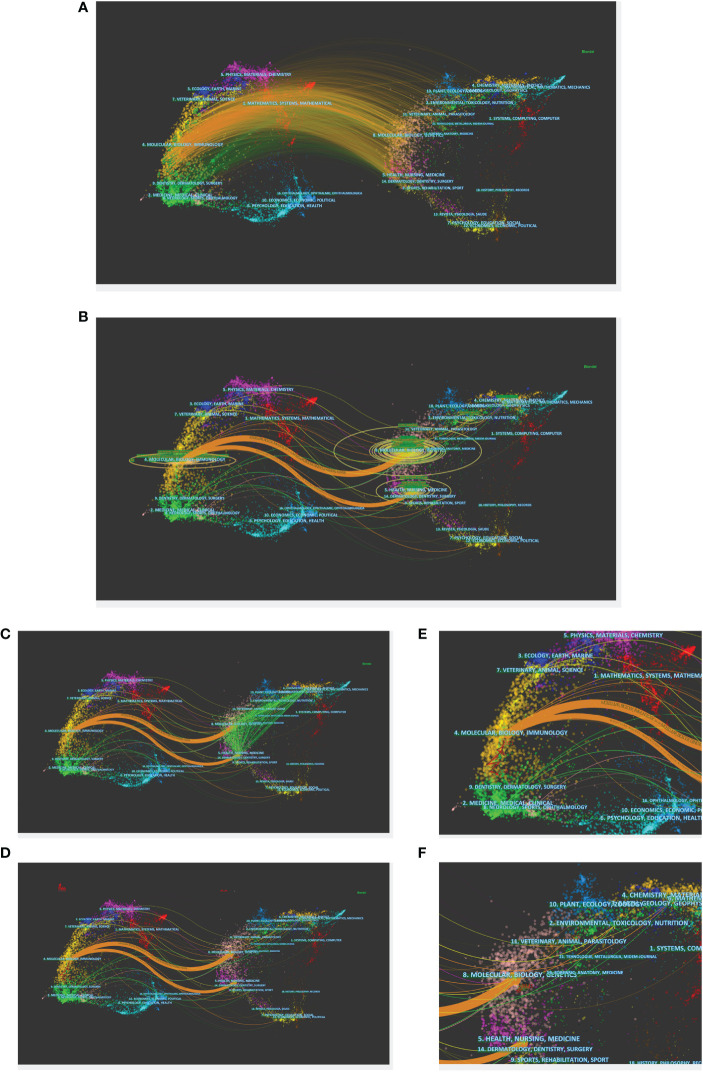
The dual-map overlay and corresponding disciplines. **(A)** The dual-map overlay of citing and cited article. Labels indicate corresponding disciplines of citing or cited articles. **(B)** The dual-map overlay using z-score function. **(C)** The co-citation links of the cited journals. **(D)** The citing and cited trajectory from 2010 to 2021. **(E, F)** The partial enlargement of the trajectory.

The dual-map overlay is an efficient tool in CiteSpace to reveal the disciplines to which the records belong. There is a base map of citing journals on the left and cited journals on the right. Citation waves are depicted on the base map, starting from the citing journals on the left and pointing to the cited side. Labels at the centroid of clusters indicate corresponding disciplines that citing or cited articles published in (**Figure 5A**). We can consider the citing side as research front over the years and the cited side as their intellectual base (21, 31).

We used the z-score function to highlight strong connections, thus improving the clarity and making them easy to recognize (**Figure 5B**). Journals with adequate volume were marked with different size of circles at the end of citation waves. In this dual map, the biggest and chief source discipline was easily recognized as (1)”Molecular, Biology, Immunology.” The disciplinary areas of (2)”Veterinary, Animal, Science,” (3)”Medicine, Medical, Clinical,” and (4)”Neurology, Sports, Ophthalmology” were also involved, but with smaller size. The majority of the citation links were directed to two disciplines: (1)”Molecular, Biology, Genetics” and (2) “Health, Nursing, Medicine.” In addition, the areas of (3) “Environmental, Toxicology, Nutrition” and (4)”Chemistry, Materials, Physics” were also compelling.

Dashed lines across different clusters depict co-citation links of the cited journals (**Figure 5C**). We can see strong boundary crossing connections between the four main disciplines stated above.

**Figures 5D–F** shows the citing and cited path with starting and ending year on the corresponding side, whose trajectory can help illustrate the dynamics of publication at disciplinary level (31). The citing trajectory presented a general shift from lower zones to upper areas on the map. Based on the citation waves, the starting point of the citing trajectory appeared to be influenced by the development of the discipline of “Medicine, Medical, Clinical,” whereas the ending point of the citing trajectory was obviously predominated by publications in “Molecular, Biology, Immunology.” It showed a rough tendency that the hotspots of research front drifted from “Medicine, Medical, Clinical” towards “Molecular, Biology, Immunology” over the past 12 years. Speaking of the cited trajectory, its theme seemed to be fixed over time, indicating that our topic is built on the top of stable intellectual base.

### 3.5 Co-occurrence analysis

**Figure 6:**
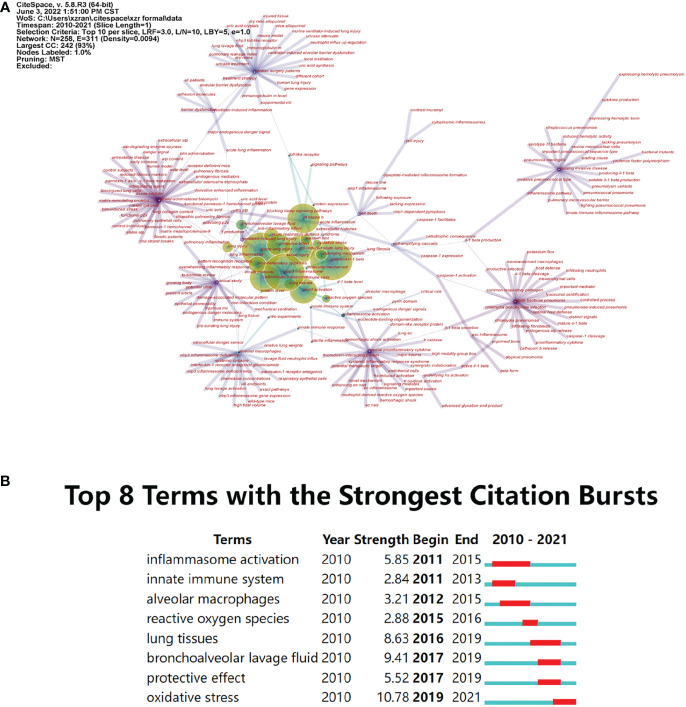
Co-occurrence analysis of terms of NLRP3 in ALI/ARDS. **(A)** The terms co-occurrence network of the 508 documents. Nodes represent noun phrases. Lines refer to co-occurring relationship. **(B)** The terms with the strongest citation bursts.

The following network in **Figure 6A** was generated based on co-occurring terms, that was, noun phrases from title, abstract, author keywords, and keywords plus. Through this overview, we can directly see the microstructure and interconnection of the domain and explore the research hot spots.

We used the pruning method, Minimum Spanning Tree, and selected the top 10 most frequent items in each year to make the network more concise and limpid. Each node represents a noun phrase. The size denotes the term’s frequency, and the tree ring can show the distribution in years. Lines between nodes refer to co-occurring relationship, and their thickness represents connection strength. The color of both nodes and lines represents the year of publication, purple the earliest and yellow the latest.

The term “acute lung injury” had the highest frequency of 208. Other frequent terms included “interleukin-1β” (183), “NLRP3 inflammasome” (170), “NF-κB” (123), and “NLRP3 activation” (98). These terms were indispensable components in this topic. Subsequently, we obtained a total of eight terms with the strongest citation bursts, which referred to fast growing topics over the years. The burst strength and time duration of these eight terms are shown in **Figure 6B**.

### 3.6 Co-citation analysis

Two papers shared a co-citation relationship when they are cited together by another document (19). CiteSpace uses this theory to construct an overview co-citation network so that we can identify important papers, the intellectual base, and the research frontiers within this field. Every node represents a reference. The tree ring showed the frequency by year, thickness represents quantity. Links between nodes reflected co-citation strength. Their color represented the year of publication (nodes) and co-citation (lines) from 2010 to 2021, purple the earliest and yellow the latest, which is universal in the following description.

**Figure 7:**
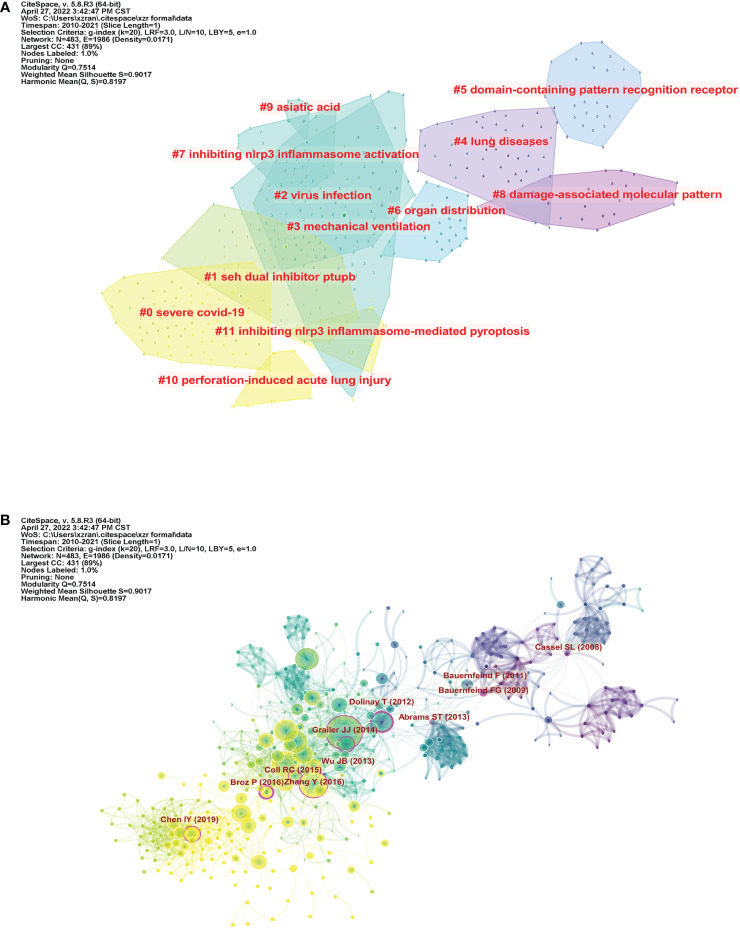
Mapping on co-cited references of NLRP3 in ALI/ARDS. **(A)** The major clusters of the co-citation network. **(B)** The co-citation network of the 508 references. Every node represents a document, and lines reflect co-citation strength. Connective papers with high centrality value are marked with purple rings.

A total of 12 clusters, each having silhouette value higher than 0.7, were synthesized based on the co-cited references and numbered from 0 to 11 by size: 0, severe COVID-19; 1, dual inhibitor PTUPB; 2, virus infection; 3, mechanical ventilation; 4, lung diseases; 5, domain-containing pattern recognition receptor; 6, organ distribution; 7, inhibiting NLRP3 inflammasome activation; 8, damage-associated molecular pattern; 9, asiatic acid; 10, perforation-induced acute lung injury; and 11 inhibiting NLRP3 inflammasome-mediated pyroptosis. We can have a better view of the structure and major areas constituting this research in **Figure 7A**.

Those with centrality value >0.1 are considered to be influential and connective between different clusters (20). These specific articles were marked with purple circles in **Figure 7B**. Dolinay’s work (2012), which explored how inflammasome pathway and correlated cytokines contributed to acute lung injury, had the highest centrality value (0.38), thus tending to have the most boundary spanning potential.

### 3.7 Burst detection

**Figure 8:**
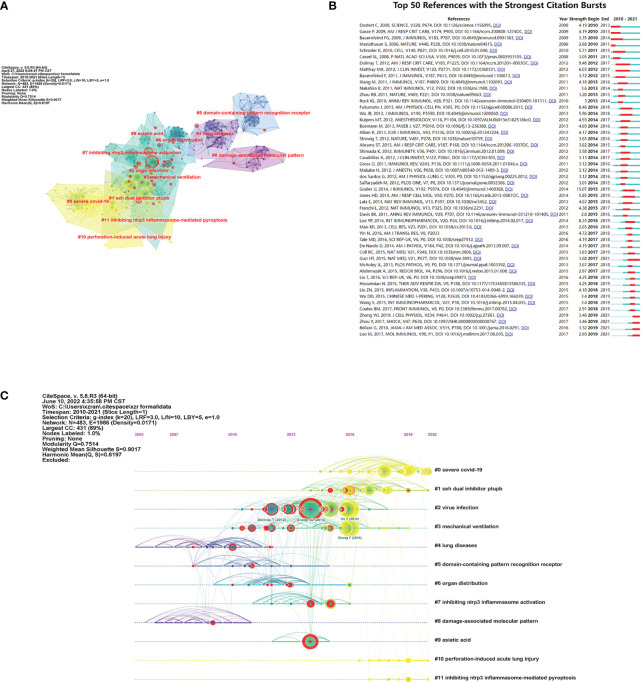
Burst detection and timeline view. **(A)** Burst references marked with red rings in the co-citation network. **(B)** Burst strength and time duration of the top 50 references with the strongest citation bursts. **(C)** Timeline view of the 12 clusters.

Citation burst means that the number of citation increases in a short period of time, so burst detection can reveal the most active areas and emerging trends in the network. As shown in **Figure 8A**, nodes with citation bursts were marked with red rings. It was identified that Cluster 2 “virus infection” included the most bursts (16 burst references), illustrating its critical role in this field’s development. Except that, Clusters 3, 1, 4, and 6 included 8, 7, 6, and 6 burst references, respectively. Details of the top 50 references with citation bursts are shown in **Figure 8B**.

In timeline view (**Figure 8C**), it is easily observed how every single cluster develops over time. In the early stage, Cluster 4 “lung diseases” and Cluster 8 “damage-associated molecular pattern” showed up first, whereas other clusters acquired more and more attention, and Cluster 2 “virus infection,” with high frequency and the most bursts, became the most active theme ever. Simultaneously, it is also worth noting that Cluster 0 “severe COVID-19” and Cluster 1 “dual inhibitor PTUPB” emerged several years ago and had the biggest size of all. Cluster 10 “perforation-induced acute lung injury” and Cluster 11 “inhibiting NLRP3 inflammasome-mediated pyroptosis” also appeared in recent years. We discovered that the spotlight and research tendency had transformed from Cluster 2 “virus infection” to Cluster 0 “severe COVID-19” and Cluster 1 “dual inhibitor PTUPB” recently.

## 4 Discussion

In this study, we collected the bibliographic data of literature on NLRP3 inflammasome in ALI/ARDS from the SCIE database and conducted a series of bibliometric analyses to explore the intellectual structure, developing trajectory and emerging trends in this field. Bibliometrix (3.1.4) R package and CiteSpace (5.8.R3) were used for deeper analysis. In general, 508 English articles and reviews published from 2010 to 2021 were identified. Although there were few articles at the beginning, the annual number of publications presented a rapidly developing trend especially in recent years. NLRP3 was largely studied over the last decade, but it was a relatively fresh topic to discuss the role of NLRP3 inflammasome in ALI/ARDS development, and it gained increasing attention from researchers. It is reported that activated NLRP3 can induce macrophage pyroptosis, therefore finally leading to ALI/ARDS, which was also a novel mechanism regarding the role of macrophages in ALI/ARDS (32, 33). The outbreak of COVID-19 and the intensive research in severe cases with ALI/ARDS may be partly responsible for the rapid increase.

Among the 42 regions and countries involved in the publication, China and the USA contributed over 85% of the literature altogether, both with high H-index and citation counts. Seven of the top 10 institutions and four of the top 10 corresponding authors belonged to China. China was the most active country and had good performance in combining quality with productivity. Meanwhile, seven of the top 15 highly cited references were delivered by American researchers, showing absolute advantage in publishing top papers. The research group from Central South University, Jilin University, LERU, and the University of Michigan made significant contributions in this field. Involving four of the 15 most highly cited papers, *The Journal of Immunology* had supreme citation count and G-index, while *International Immunopharmacology* grew rapidly in the past 3 years and obviously had the most publications in this field.

The major disciplines of research front over the past 12 years underwent a general transformation from “Medicine, Medical, Clinical” to “Molecular, Biology, Immunology.” “Molecular, Biology, Immunology” took the biggest and chief source disciplines overall. The major discipline of intellectual base, “Molecular, Biology, Genetics” and “Health, Nursing, Medicine,” remained stable over the years and closely associated with each other.

In the co-occurring network, we can conclude that except for “acute lung injury” and “NLRP3 inflammasome,” “interleukin 1β,” “NF-κB,” and “NLRP3 activation” occurred most frequently, indicating indivisible relationship with this topic. This is reasonable because NLRP3 inflammasome acts as a crucial role in controlling the maturation of proinflammatory IL-1β, and NF-κB pathway is also a key member regarding regulating IL-1 maturation and the activation of NLRP3 inflammasome (34–36). The term of fast-growing topic with the strongest citation burst was “oxidative stress” (Strength 10.78), which is an essential pathological process in ALI/ARDS (37) and also the youngest burst term beginning at 2019. In the process of oxidative stress, NF-κB pathway can trigger the inflammatory response, and the activated NLRP3 inflammasome can lead to more production of reactive oxygen species (ROS) (37).

In the co-citation network, the cited references were divided into 12 clusters of different years, sizes, and themes. We can find which cluster was largely investigated and which one interacted closely with others. At the beginning, researchers mainly focused on two topics: “lung diseases” and “damage-associated molecular pattern.” Then, the themes of cited papers bloomed. Several clusters emerged successively and possessed a lot of burst references, and the line of development went from “domain-containing pattern recognition receptor” to “organ distribution,” then to “virus infection” and “mechanical ventilation,” finally to “inhibiting NLRP3 inflammasome activation” and “asiatic acid.” However, in recent years, new topics have emerged and now are still watched, which are “severe COVID-19,” “dual inhibitor PTUPB,” “perforation-induced acute lung injury,” and “inhibiting NLRP3 inflammasome-mediated pyroptosis.” The former two were also the biggest two clusters and worth paying attention to, and the latter two were small groups. Before therapeutic inhibitors of NLRP3 inflammasome were available, biological IL-1β inhibitors were used to neutralize IL-1β and attenuate NLRP3-induced inflammation (38). Antagonists and therapies that specifically target NLRP3 inflammasome were widely studied (38). It was found that melatonin can inhibit the release of extracellular histones and block histone-induced NLRP3 inflammasome activation directly, therefore alleviating ALI (39). Ly6G+ neutrophil-derived miR-223 was found to target the expression of NLRP3 inflammasome by activating a negative feedback pathway (40). Among the 12 co-citation clusters, “virus infection” was a focal point and the most active area because it contained the largest number of burst references. Meanwhile, “dual inhibitor PTUPB,” “mechanical ventilation,” “lung diseases,” and “organ distribution” also received plenty of popularity.

Since the outbreak of COVID-19 in December 2019, which was caused SARS-CoV-2, COVID-19-associated ALI/ARDS has been widely discussed, and the role of NLRP3 inflammasome in its progressing has received great attention. Although it is often manifested as mild symptoms, the rapid progress of ARDS with systematic inflammation in severe cases especially those with complications made it fatal (41). Although Cluster 0 “severe COVID-19” was relatively new, it had the biggest number of members and received broad attention from researchers. It may contribute to the rapidly increasing trend of the annual number of publications over the past few years as presented above (**Figures 2**, **8C**), illustrating the great significance in the mechanism and treatment of COVID-19-associated ALI/ARDS.

Recently, direct evidence has been provided that inflammasome activation exists in progressing COVID-19, such as in blood monocytes and lung tissues along with the occurrence of pyroptosis (42–45). While NLRP3 inflammasome mediates IL-1β maturation, IL-1β can also affect the activation of NLRP3 inflammasome. SARS-CoV-2 infection can result in multiple cytokines expression including IL-1β, which plays a key role in the pathogenesis of virus infection associated ALI/ARDS, mediating acute proinflammatory cascade after infection and therefore leading to a series of proinflammatory responses including the activation of NLRP3 (12, 46). It is shown that SARS-CoV3a protein can activate NLRP3 inflammasome by inducing the secretion of IL-1β (47). Although the overactivation of NLRP3 inflammasome and release of cytokines can cause tissue and organ damage, it is demonstrated that inflammasome and IL-1β can also provide protections in the acute phase of multiple pathogens infection (48). Studies are needed to explore whether this protective role is played in the early phase of SARS-CoV-2 infection, therefore prompting us to pay more attention to the timing of inflammasome targeted therapies (44). In addition, NLRP3 may be one of the reasons why COVID-19 is more likely to affect older people, as it manifests an increase of activity during the aging process (30). The critical role of NLRP3 inflammasome in ALI/ARDS of different causes including COVID-19 makes it worthwhile to investigate the detailed mechanisms and targeting therapies.

In this study, we focused on depicting the characteristics and searching for emerging trends of the literature data about NLRP3 inflammasome in ALI/ARDS from 2010 to 2021 from the SCIE database, using CiteSpace (5.8.R3) for visualization. Although related themes of bibliometric studies were conducted, including inflammasome in neurological diseases (49–51), there is no bibliometric analysis focusing on NLRP3 inflammasome in ALI/ARDS so far. Chen Wang’s work (2020) (52) retrieved a broad range of literature on ALI/ARDS during 2009–2019 from WOS, exploring the distribution of literature contribution and analyzing the trends of this specific disease in a wide dimension. They considered the keywords “Berlin definition,” “stromal cells,” and “protects” to be the potential hot spots in the discipline of ALI/ARDS. Compared with Chen Wang’s work, our study chose a more focalized topic with obvious rapid growth trend, also revealing more precise emerging trends for guiding future research through visualization by CiteSpace software. Furthermore, Sheng Wang et al. (33) analyzed the research hot spots and frontiers of literature about macrophages in ALI, also mentioning NLRP3 inflammasome as a novel mechanism regarding the role of macrophages in ALI/ARDS, which can support the results of this research to some extent. These two studies focused on different topics. In addition, compared with Sheng Wang’s work, our study investigated from more and deeper angles including the trends of journals’ contribution, citation count variation of landmark articles, and trajectory of disciplines and references with citation bursts. Furthermore, we provided a more detailed co-citation analysis using CiteSpace software. Nevertheless, this study has some limitations. First, we considered only literature written in English, so papers in other languages were not included. Second, we considered only literature collected in WoSCC, so some papers in other databases such as Scopus and Embase were not taken into account. Lastly, some latest studies may not be included due to the rapid update of database.

## 5 Conclusion

This bibliometric analysis reveals a rapid growth trend of the relatively novel topic: NLRP3 inflammasome in ALI/ARDS. China was the largest contributor, while the USA offered the most landmark papers in this field. The major disciplines of research front have drifted from “Medicine, Medical, Clinical” to “Molecular, Biology, Immunology.” Studies about oxidative stress became a hot spot in recent years. Virus-infection-induced ALI/ARDS was an active area of research, and with the epidemic outbreak of COVID-19, research on the role and therapeutic potential of NLRP3 inflammasome in COVID-19-associated ALI/ARDS was emerging as a hot topic, which is still necessary to explore in the future.

## Data availability statement

The raw data supporting the conclusions of this article will be made available by the authors, without undue reservation.

## Author contributions

ZX and SW conceived the study. ZX, SH, WX, and WM were involved in the data collection and analysis. ZX and JW wrote the manuscript. HD, HY, WZ, QL, HZ, and XL revised the manuscript. ZX, SH, and WX contributed equally to this work. All authors contributed to the article and approved the submitted version.
